# Supplementary material for: Characterization and Risk Assessment of PM2.5-Bound Polycyclic Aromatic Hydrocarbons and Their Derivatives Emitted from a Typical Pesticide Factory in China
Source: Toxics. 2023 Jul 23;11(7):637. doi: 10.3390/toxics11070637 (PMC10385953; doi:10.3390/toxics11070637)
Supplement: Supplementary file 1 [file toxics-11-00637-s001.zip › toxics-2495147-supplementary.pdf]

Supplementary Materials: Characterization and Risk Assessment of $PM_{2.5}$-Bound Polycyclic Aromatic Hydrocarbons and their Derivatives Emitted from a Typical Pesticide Factory in China

Diwei Wang [1,2], Shengmin Wu [2,*], Xuesong Gong [3,*], Tao Ding [2], Yali Lei [4], Jian Sun [1] and Zhenxing Shen [1]

[1] Department of Environmental Science and Engineering, Xi'an Jiaotong University, Xi'an 710049, China
[2] The State Key Laboratory of Environmental Assessment and Pollution Control of Pesticides for Environmental Protection, Nanjing Institute of Environmental Sciences, Ministry of Ecological Environment, Nanjing 210042, China; dingtao@xpu.edu.cn
[3] School of Environmental and Chemical Engineering, Xi'an Polytechnic University, Xi'an 710048, China
[4] Key Lab of Geographic Information Science of the Ministry of Education, School of Geographic Sciences, East China Normal University, Shanghai 200241, China; yllei@geo.ecnu.edu.cn
* Correspondence: wsm@nies.org (S.W.); xsgong@xpu.edu.cn (X.G.)

Contents:

Text S1. The risk assessment of exposure to $PM_{2.5}$-bound PAHs emission from pesticide factory.

Table S1. The meteorological parameters, including temperature (T), relative humidity (RH), wind speed (WS), and wind direction (WD) during the sampling period.

Table S2. Individual profile of detected PAHs in this study.

Table S3. Toxicity equivalent factor (TEF) and mutagenic potency factor (MEF) values for individual PAHs species.

Table S4. Parameters used in the incremental lifetime cancer risk (ILCR) estimation.

Table S5. The average concentration of individual PAHs species (ng/m^3).

Table S6. The TEQ and MEQ of individual PAHs species during the sampling period.

Figure S1. The map of the sampling area. The red star indicates the target pesticide factory, and the blue ball indicates the sampling point. The map was obtained on the website https://www.tianditu.gov.cn/. Last access date: 15 July 2023.

Text S1. The risk assessment of exposure to $PM_{2.5}$-bound PAHs emission from pesticide factory.

1. Total carcinogenic equivalent toxicity (TEQ)

The TEQ was calculated according to PAHs concentrations and toxicity equivalent factor (TEF), and following equation (1):

$$\Sigma TEQ = \Sigma C_i \times TEF_i \quad (1)$$

where Ci is the i-th PAHs concentration (ng/m^3); TEF_i are the TEF of the i-th PAHs. TEF values for individual PAHs were obtained from previous literature [1–3]. In this study, a total of 28 TEF of PAHs were used, including 18 p-PAHs, 1 a-PAHs, 1 o-PAHs and 8 n-PAHs, as summarized in Table S3.

2. Total mutagenic equivalent toxicity (MEQ)

The MEQ was calculated according to PAHs concentrations and mutagenic potency factor (MEF), and following equation (2):

$$\Sigma MEQ = \Sigma C_i \times MEF_i \quad (2)$$

where C_i is the i-th PAHs concentration (ng/m^3); MEF_i are the MEF of the i-th PAHs. As summarized in Table S3, a total of 8 MEF of p-PAHs were found in previous study [4] and were used in present study.

3. Incremental lifetime cancer risk (ILCR)

The ILCR caused by inhalation, ingestion and dermal contact was calculated using the United States Environmental Protection Agency (US EPA) standard models [5,6] as following equation (3)–(6):

$$ILCRs_{Inhalation} = \frac{CS \times (CSF_{Inhalation} \times \sqrt[3]{(\frac{BW}{70})}) \times IR_{Inhalation} \times EF \times ED}{BW \times AT \times PEF} \quad (3)$$

$$ILCRs_{Ingestion} = \frac{CS \times (CSF_{Ingestion} \times \sqrt[3]{(\frac{BW}{70})} \times IR_{Ingestion} \times EF \times ED}{BW \times AT \times 10^6} \quad (4)$$

$$ILCRs_{Dermal} = \frac{CS \times (CSF_{Dermal} \times \sqrt[3]{(\frac{BW}{70})}) \times SA \times AF \times ABS \times EF \times ED}{BW \times AT \times 10^6} \quad (5)$$

$$ILCR = ILCR_{Inhalation} + ILCR_{Ingestion} + ILCR_{Dermal} \quad (6)$$

where CS is the sum of converted $PM_{2.5}$-bound PAHs concentrations based on the TEF (mg kg^{-1}), CSF is carcinogenic slope factor (mg kg^{-1} d^{-1})$^{-1}$, BW is body weight (kg), $IR_{Inhalation}$ is the inhalation rate (m^3 d^{-1}), $IR_{Ingestion}$ is the soil intake rate (mg d^{-1}), EF is the exposure frequency (d $year^{-1}$), ED is the exposure duration (year), AT is the average life span (year), PEF is the soil dust produce factor (m^3 kg^{-1}), SA is the dermal surface exposure (cm^2 d^{-1}), AF is the dermal adherence factor (mg cm^{-2}), ABS is the dermal adsorption fraction. All the used parameters in the ILCR model in present study are listed in Table S4.

4. Loss of life expectancy (LLE)

LLE is the loss in expectation of life caused by carcinogenic risk, which is a new environmental health risk assessment technology that normalizes and compares the carcinogenic risk and non-carcinogenic risk caused by environmental pollution [7]. Previous studies have shown that the LLE equivalent of adults corresponding to the 10^{-5} of ILCR caused by pollution exposure was 51.2 min [7]. The LLE caused by carcinogenesis of PAHs was calculated as following equation (7):

$$LLE = 51.2 \times (ILCR/10^{-5}) \quad (7)$$

Table S1. The meteorological parameters, including temperature (T), relative humidity (RH), wind speed (WS), and wind direction (WD) during the sampling period.

Date	T (°C)	RH (%)	WD	WS (m/s)
2023.3.15 day	5~14	70	Northeast	7
2023.3.15 night	6~14	75	East	5
2023.3.16 day	4~13	90	East	5
2023.3.16 night	4~13	83	East	5
2023.3.17 day	1~9	87	Northeast	4
2023.3.17 night	1~9	68	Northeast	1
2023.3.18 day	−1~15	70	Northwest	2
2023.3.18 night	1~15	70	Southwest	1
2023.3.19 day	2~17	70	Southeast	2
2023.3.19 night	2~17	62	Southeast	2
2023.3.20 day	3~18	66	Southeast	3
2023.3.20 night	3~18	76	Southeast	2
2023.3.21 day	7~18	84	East	3
2023.3.21 night	7~18	84	Northeast	3
2023.3.22 day	10~14	95	Northeast	3
2023.3.22 night	10~14	94	North	4
2023.3.23 day	8~17	45	Northeast	6
2023.3.23 night	8~17	35	East	4

Table S2. Individual profile of detected PAHs in this study.

	PAHs species	Abbreviation	Molar weight (g/mol)	Rings
Parent-PAHs	acenaphthylene	ACY	152.20	2
	acenaphthene	ACE	154.21	2
	fluorene	FLO	166.22	2
	phenanthrene	PHE	178.23	3
	anthracene	ANT	178.23	3
	fluoranthene	FLA	202.26	4
	pyrene	PYR	202.25	4
	benzo[a]anthracene	BaA	228.29	4
	chrysene	CHR	228.29	4
	benzo[b]fluoranthene	BbF	252.31	4
	benzo[j+k]fluoranthene	BkF	252.31	4
	benzo[a]fluoranthene	BaF	252.31	4
	benzo[e]pyrene	BeP	252.31	5
	benzo[a]pyrene	BaP	252.31	5
	perylene	PER	252.31	5
	indeno[1,2,3-cd]pyrene	IcdP	276.33	5
	dibenzo[a,h]anthracene	DBahA	278.35	5
	benzo[ghi]perylene	BghiP	276.33	6
	coronene	COR	300.35	6
	dibenzo[a,e]pyrene	DBaeP	302.37	6
	cyclopenta[cd]pyrene	CPcdP	226.27	4
	picene	PIC	278.35	5
Alkylated-PAHs	2-methylnaphthalene	2M-NAP	142.20	2
	1-methylnaphthalene	1M-NAP	142.20	2
	2,6-dimethylnaphthalene	2,6DM-NAP	156.22	2
	9-methylanthracene	9M-ANT	192.26	3
	methylfluoranthene	M-FLA	216.28	3
	retene	RET	234.34	3
	methylchrysene	M-CHR	242.31	4
Oxygenated-PAHs	1,4-naphthoquinone	1,4-NAQ	158.15	2
	1-naphthaldehyde	1-NAA	156.18	2
	1-acenaphthenone	1-ACO	168.19	2
	9-fluorenone	9-FO	180.20	2
	9,10-anthraquinone	9,10-ATQ	208.21	3
	1,8-naphthalic anhydride	1,8-NAA	198.17	3
	benzo(a)anthracene-7,12-dione	BaAQ	258.27	4
	1,4-chrysenequinone	1,4-CHRQ	258.27	4
	5,12-naphthacenequinone	5,12-NAAQ	258.27	4
	6H-benzo(c,d)pyrene-6-one	6H-BcdPO	254.28	5
	benzanthrone	BZA	230.26	4
Nitrated-PAHs	2-nitrobiphenyl	2N-BIP	199.20	2
	5-nitroacenaphthene	5N-ACE	199.21	2
	2-nitrofluorene	2N-FLO	211.22	2
	9-nitrophenanthrene	9N-PHE	223.23	3
	9-nitroanthracene	9N-ANT	223.23	3

3-nitrofluoranthene	3N-FLA	247.25	4
1-nitropyrene	1N-PYR	247.26	4
2,7-dinitrofluorene	2,7DN-FLO	256.21	2
6-nitrochrysene	6N-CHR	273.29	4
1,3-dinitropyrene	1,3-DNP	292.26	4
1,6-dinitropyrene	1,6-DNP	292.26	4
6-nitrobenzo(a)pyrene	6N-BaP	297.31	5

Table S3. Toxicity equivalent factor (TEF) and mutagenic potency factor (MEF) values for individual PAHs species.

PAHs species	Abbreviation	TEF	MEF
Acenaphthylene	ACY	0.001[2]	-
Acenaphthene	ACE	0.001[2]	-
Fluorene	FLO	0.001[2]	-
Phenanthrene	PHE	0.001[2]	-
Anthracene	ANT	0.01[2]	-
Fluoranthene	FLA	0.001[2]	-
Pyrene	PYR	0.001[2]	-
Benzo[a]Anthracene	BaA	0.1[2]	0.082[4]
Chrysene	CHR	0.01[2]	0.017[4]
Benzo[b]Fluoranthene	BbF	0.1[2]	0.25[4]
Benzo[k]Fluoranthene	BkF	0.1[2]	0.11[4]
Benzo[e]Pyrene	BeP	0.002[1]	-
Benzo[a]Pyrene	BaP	1[2]	1[4]
Indeno[1,2,3-cd]Pyrene	IcdP	0.1[2]	0.31[4]
Dibenzo[a, h]Anthracene	DBahA	5[2]	0.29[4]
Benzo[g, h, i]Perylene	BghiP	0.01[2]	0.19[4]
Dibenzo[a, e]Pyrene	DBaeP	2.9[1]	-
2-Methylnaphthalene	2M-NAP	0.001[2]	-
Cyclopenta[cd]Pyrene	CPcdP	6.9[1]	-
6H-Benzo(c, d)Pyrene-6-One	BcdPO	0.32[1]	-
5-Nitroacenaphthene	5N-ACE	0.01[3]	-
2-Nitrofluorene	2N-FLO	0.01[3]	-
9-Nitroanthracene	9N-ANT	0.0032[1]	-
3-Nitrofluoranthene	3N-FLA	0.0026[1]	-
1-Nitropyrene	1N-PYR	0.1[1]	-
6-Nitrochrysene	6N-CHR	10[3]	-
1,3-Dinitropyrene	1,3-DNP	0.031[1]	-
1,6-Dinitropyrene	1,6-DNP	0.28[1]	-

"-" represents not available.

Table S4. Parameters used in the incremental lifetime cancer risk (ILCR) estimation.

Parameters	Male adult	Female adult	References
Body weight (BW) (kg)	71.4	63	[8]
$CSF_{Inhalation}$ $(mg·kg^{-1}·d^{-1})^{-1}$	3.85	3.85	[6]
$CSF_{Infestion}$ $(mg·kg^{-1}·d^{-1})^{-1}$	7.3	7.3	[6]
CSF_{Dermal} $(mg·kg^{-1}·d^{-1})^{-1}$	25	25	[6]
Inhalation rate ($IR_{Inhalation}$) ($m^3·d^{-1}$)	18.7	15.1	[8]
Soil intake rate ($IR_{Ingestion}$) ($m^3·d^{-1}$)	100	100	[9]
Exposure frequency (EF) ($d·a^{-1}$)	180	180	[10]
Exposure duration (ED)(a)	30	30	[8]
Averaging life span (AT) (d)	25550	25550	[11]
Dermal exposure area (SA) (cm^2)	5700	5700	[9]
Dermal adherence factor (AF) ($mg·cm^{-2}$)	0.07	0.07	[9]
Dermal adsorption fraction (ABS)	0.13	0.13	[9]
Particle emission factor (PEF) ($m^3·kg^{-1}$)	$1.36·10^9$	$1.36·10^9$	[9]

Table S5. The average concentration of individual PAHs species (ng/m^3).

	PAHs species	Daily (n = 17)	Daytime (n = 9)	Nighttime (n = 8)
p-PAHs	ACY	4.32±3.27	4.11±3.81	4.56±2.78
	ACE	1.71±1.04	1.81±1.28	1.61±0.76
	FLO	3.31±2.32	3.56±3.00	3.03±1.36
	PHE	2.69±2.34	2.74±2.85	2.64±1.79
	ANT	1.29±0.67	1.36±0.84	1.21±0.45
	FLA	6.80±5.68	7.07±6.98	6.51±4.21
	PYR	9.04±7.52	9.66±9.30	8.34±5.40
	BaA	4.24±4.11	4.05±4.15	4.44±4.34
	CHR	8.21±5.46	8.63±7.03	7.75±3.37
	BbF	6.31±4.46	6.15±5.21	6.49±3.80
	BkF	6.27±6.41	6.28±7.6	6.26±5.29
	BaF	3.16±2.60	3.22±3.14	3.10±2.03
	BeP	8.47±7.87	9.05±9.95	7.82±5.25
	BaP	7.58±5.41	7.76±6.44	7.36±4.41
	PER	3.77±4.96	3.52±5.32	4.04±4.87
	IcdP	3.57±2.44	3.63±2.75	3.51±2.23
	DBahA	4.71±3.41	4.78±3.68	4.64±3.32
	BghiP	9.66±9.15	8.75±8.40	10.68±10.43
	COR	5.95±5.45	5.85±6.43	6.06±4.53
	DBaeP	3.52±2.74	3.56±3.25	3.49±2.26
	CPcdP	2.08±1.76	2.06±2.03	2.10±1.53
	PIC	5.89±3.77	5.80±4.40	5.98±3.21
Σp-PAHs		112.55±89.69	113.39±105.97	111.61±74.52
a-PAHs	2M-NAP	2.61±1.88	2.79±2.28	2.4±1.44
	1M-NAP	1.24±1.04	1.32±1.34	1.15±0.66
	2,6DM-NAP	4.13±3.11	4.71±3.97	3.48±1.80
	9M-ANT	3.31±3.00	3.71±3.92	2.86±1.60
	M-FLA	1.75±1.97	1.75±2.35	1.76±1.60
	RET	3.04±1.86	3.12±2.28	2.95±1.40
	M-CHR	1.96±1.55	1.96±2.00	1.95±0.95
Σa-PAHs		18.05±13.76	19.37±17.57	16.56±8.66
o-PAHs	1,4-NAQ	1.84±1.07	1.98±1.28	1.70±0.85
	1-NAA	1.58±1.12	1.65±1.39	1.49±0.79
	1-ACO	1.08±0.92	1.13±1.11	1.02±0.71
	9-FO	11.79±8.94	11.81±11.3	11.77±6.05
	9,10-ATQ	10.68±8.93	10.96±11.02	10.36±6.59
	1,8-NAA	4.02±3.67	4.12±4.62	3.92±2.51
	BaAQ	9.37±9.17	9.29±11.27	9.47±6.87
	1,4-CHRQ	5.88±4.01	6.33±5.28	5.37±2.07
	5,12-NAAQ	5.45±4.44	5.66±5.60	5.20±3.03
	6H-BcdPO	7.35±7.43	7.29±8.67	7.42±6.34
	BZA	7.08±6.28	6.98±7.93	7.20±4.26
Σo-PAHs		66.13±54.79	67.20±68.85	64.92±37.94
n-PAHs	2N-BIP	0.45±0.24	0.45±0.29	0.45±0.18
	5N-ACE	0.27±0.13	0.28±0.17	0.26±0.09
	2N-FLO	0.20±0.13	0.22±0.17	0.18±0.08
	9N-PHE	0.15±0.08	0.15±0.10	0.15±0.07

9N-ANT	0.96±0.74	1.00±0.96	0.92±0.45
3N-FLA	0.30±0.15	0.31±0.18	0.30±0.12
1N-PYR	0.29±0.18	0.29±0.19	0.30±0.17
2,7DN-FLO	0.26±0.16	0.27±0.19	0.25±0.13
6N-CHR	0.15±0.09	0.15±0.11	0.14±0.06
1,3-DNP	0.26±0.14	0.26±0.16	0.25±0.13
1,6-DNP	0.29±0.17	0.29±0.18	0.29±0.16
6N-BaP	0.33±0.18	0.33±0.21	0.32±0.15
Σn-PAHs	3.90±2.24	4.00±2.79	3.80±1.61

Table S6. The TEQ and MEQ of individual PAHs species during the sampling period.

PAHs species	TEQ (ng/m^3)	MEQ (ng/m^3)
ACY	0.004±0.003	-
ACE	0.002±0.001	-
FLO	0.003±0.002	-
PHE	0.003±0.002	-
ANT	0.013±0.007	-
FLA	0.007±0.006	-
PYR	0.009±0.008	-
BaA	0.424±0.411	0.347±0.337
CHR	0.082±0.055	0.14±0.093
BbF	0.631±0.446	1.578±1.116
BkF	0.627±0.641	0.689±0.705
BeP	0.017±0.016	-
BaP	7.575±5.41	7.575±5.41
IcdP	0.357±0.244	1.107±0.756
DBahA	23.556±17.039	1.366±0.988
BghiP	0.097±0.092	1.836±1.739
DBaeP	10.222±7.955	-
CPcdP	14.327±12.131	-
2M-NAP	0.003±0.002	-
BcdPO	2.352±2.376	-
5N-ACE	0.003±0.001	-
2N-FLO	0.002±0.001	-
9N-ANT	0.003±0.002	-
3N-FLA	0.001±0.0004	-
1N-PYR	0.029±0.018	-
6N-CHR	1.489±0.908	-
1,3-DNP	0.008±0.004	-
1,6-DNP	0.08±0.047	-

"-" represents not available.

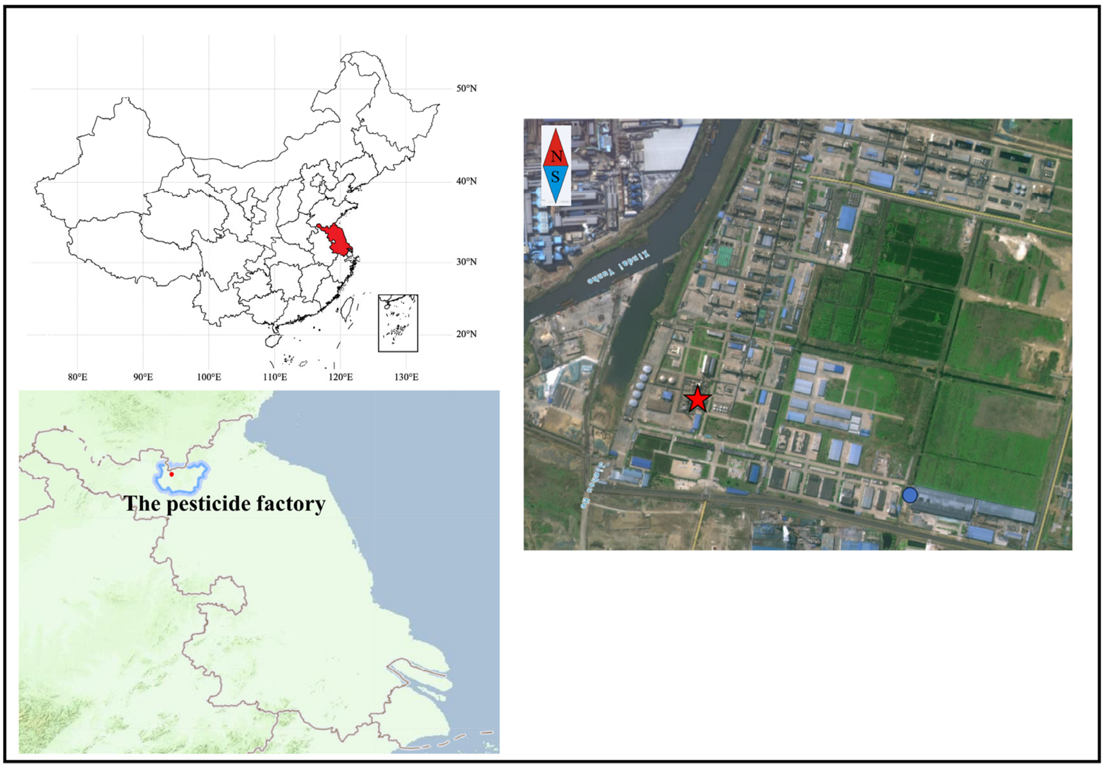

Fig. S1 The map of the sampling area. The red star indicates the target pesticide factory, and the blue ball indicates the sampling point. The map was obtained on the website https://www.tianditu.gov.cn/. Last access date: 15 July 2023.

Reference:

1. Durant, J.L.; Busby, W.F., Jr.; Lafleur, A.L.; Penman, B.W.; Crespi, C.L. Human cell mutagenicity of oxygenated, nitrated and unsubstituted polycyclic aromatic hydrocarbons associated with urban aerosols. *Mutat. Res. Genet. Toxicol.* **1996**, *371*, 123–157.
2. Nisbet, I.C.; Lagoy, P.K. Toxic equivalency factors (TEFs) for polycyclic aromatic hydrocarbons (PAHs). *Regul. Toxicol. Pharmacol.* **1992**, *16*, 290–300.
3. Wei, C.; Han, Y.; Bandowe, B.A.; Cao, J.; Huang, R.J.; Ni, H.; Tian, J.; Wilcke, W. Occurrence, gas/particle partitioning and carcinogenic risk of polycyclic aromatic hydrocarbons and their oxygen and nitrogen containing derivatives in Xi'an, central China. *Sci. Total Environ.* **2015**, *505*, 814–822.
4. Jung, K.H.; Yan, B.; Chillrud, S.N.; Perera, F.P.; Whyatt, R.; Camann, D.; Kinney, P.L.; Miller, R.L. Assessment of benzo(a)pyrene-equivalent carcinogenicity and mutagenicity of residential indoor versus outdoor polycyclic aromatic hydrocarbons exposing young children in New York City. *Int. J. Environ. Res. Public Health* **2010**, *7*, 1889–1900.
5. Chen, S.C.; Liao, C.M. Health risk assessment on human exposed to environmental polycyclic aromatic hydrocarbons pollution sources. *Sci. Total Environ.* **2006**, *366*, 112–123.
6. Peng, C.; Chen, W.; Liao, X.; Wang, M.; Ouyang, Z.; Jiao, W.; Bai, Y. Polycyclic aromatic hydrocarbons in urban soils of Beijing: Status, sources, distribution and potential risk. *Environ. Pollut.* **2011**, *159*, 802–808.
7. Guo, X.; Chen, F.; Lu, J.; Zhang, W. Seasonal variation, sources, and risk assessment of $PM_{2.5}$-bound PAHs in Nantong, China: A pre- and post-COVID-19 case study. *Front. Environ. Sci.* **2022**, *10*, 947705.
8. An, Y.Q.; Hao, H.Y; Jin, H.; Yuan, S.H.; Pei, X.K.; Yang, N.J.; Liu, Y.G. Pollution level and health risk assessment of polycyclic aromatic hydrocarbons in $PM_{2.5}$ of four cities in Hebei Province. *Occup and Health*. **2018**, *34*, 3129-3133. (In Chinese)
9. Wang, W.; Huang, M.J.; Kang, Y.; Wang, H.S.; Leung, A.O.; Cheung, K.C.; Wong, M.H. Polycyclic aromatic hydrocarbons (PAHs) in urban surface dust of Guangzhou, China: Status, sources and human health risk assessment. *Sci. Total Environ.* **2011**, *409*, 4519-4527.
10. Ferreira-Baptista, L.; De Miguel, E. Geochemistry and risk assessment of street dust in Luanda, Angola: A tropical urban environment. *Atmos. Environ.* **2005**, *39*, 4501-4512.
11. Sun, J.; Shen, Z.; Zhang, T.; Kong, S.; Zhang, H.; Zhang, Q.; Niu, X.; Huang, S.; Xu, H.; Ho, K.F.; et al. A comprehensive evalua-tion of $PM_{2.5}$-bound PAHs and their derivative in winter from six megacities in China: Insight the source-dependent health risk and secondary reactions. *Environ. Int.* **2022**, *165*, 107344.
